# Effect of Probiotics on Metabolic Outcomes in Pregnant Women with Gestational Diabetes: A Systematic Review and Meta-Analysis of Randomized Controlled Trials

**DOI:** 10.3390/nu9050461

**Published:** 2017-05-05

**Authors:** Bonnie L. Taylor, Georgia E. Woodfall, Katherine E. Sheedy, Meggan L. O’Riley, Kelsie A. Rainbow, Elsa L. Bramwell, Nicole J. Kellow

**Affiliations:** Be Active Sleep & Eat (BASE) Facility, Department of Nutrition, Dietetics & Food, Monash University, Notting Hill, VIC 3168, Australia; bltay9@student.monash.edu (B.L.T.); gewoo1@student.monash.edu (G.E.W.); keshe5@student.monash.edu (K.E.S.); mlori1@student.monash.edu (M.L.O.); karia3@student.monash.edu (K.A.R.); elbra3@student.monash.edu (E.L.B.)

**Keywords:** probiotics, gut microbiota, Gestational Diabetes Mellitus, pregnancy, insulin resistance

## Abstract

The metabolic effects of probiotic administration in women with gestational diabetes mellitus (GDM) is unknown. The objective of this review was to investigate the effect of probiotics on fasting plasma glucose (FPG), insulin resistance (HOMA-IR) and LDL-cholesterol levels in pregnant women diagnosed with GDM. Seven electronic databases were searched for RCTs published in English between 2001 and 2017 investigating the metabolic effects of a 6–8 week dietary probiotic intervention in pregnant women following diagnosis with GDM. Eligible studies were assessed for risk of bias and subjected to qualitative and quantitative synthesis using a random effects model meta-analyses. Four high quality RCTs involving 288 participants were included in the review. Probiotic supplementation was not effective in decreasing FBG (Mean Difference = −0.13; 95% CI −0.32, 0.06, *p* = 0.18) or LDL-cholesterol (−0.16; 95% CI −0.45, 0.13, *p* = 0.67) in women with GDM. However, a significant reduction in HOMA-IR was observed following probiotic supplementation (−0.69; 95% CI −1.24, −0.14, *p* = 0.01). There were no significant differences in gestational weight gain, delivery method or neonatal outcomes between experimental and control groups, and no adverse effects of the probiotics were reported. Probiotic supplementation for 6–8 weeks resulted in a significant reduction in insulin resistance in pregnant women diagnosed with GDM. The use of probiotic supplementation is promising as a potential therapy to assist in the metabolic management of GDM. Further high quality studies of longer duration are required to determine the safety, optimal dose and ideal bacterial composition of probiotics before their routine use can be recommended in this patient group.

## 1. Introduction

The gut microbiota is a collective term used to refer to the microorganisms colonizing the human gastrointestinal tract [1]. It is an important ecosystem consisting of both residential and pathogenic bacteria [2,3]. Residential intestinal microbes coexist in a symbiotic relationship with their host by extracting energy from dietary components which humans lack the enzymes to digest. In return, the microbiota produce bioactive compounds shown to benefit host metabolism. Manipulation of the gut microbiome and its fermentation by-products is emerging as a promising therapeutic treatment strategy for many chronic medical conditions [4]. A variety of factors influence the gut microbiome, including host genetics, illness, antibiotic use, dietary patterns, weight loss and pregnancy [5,6,7,8]. Perturbations in the composition of the gut microbiota have been hypothesized to contribute to the pathogenesis of obesity, inflammation and insulin resistance [9].

Throughout pregnancy the gut microbiota undergoes significant changes. From the first (T1) to the third trimester (T3), the species richness of the gut microbiome decreases [8], although this has not been observed in all studies [10]. There is an increase in Proteobacteria and Actinobacteria phyla and a reduction in beneficial bacterial species *Roseburia intestinalis* and *Faecalibacterium prausnitzii* [8,11]. These changes in gut microbial composition cause inflammation and correlate with increases in fat mass, blood glucose, insulin resistance and circulating pro-inflammatory cytokines in the expectant mother [12]. This “diabetic-like” state observed during the later stages of all healthy pregnancies is thought to maximize nutrient provision to the developing fetus [13]. However, increased insulin resistance combined with an inability to secrete the additional insulin required to maintain glucose homeostasis can result in the development of gestational diabetes mellitus (GDM) in the mother and macrosomia in the baby.

GDM is defined as the development of glucose intolerance, with first onset during pregnancy [14]. This condition is associated with adverse maternal and infant health outcomes during gestation, childbirth and postpartum. Maternal comorbidities include pre-eclampsia and increased risk of infection throughout pregnancy [15]. A seven-fold increased risk of the mother developing type 2 diabetes (T2DM) postpartum has also been reported [16]. Infant morbidity includes risk of fetal malformations and diabetic fetopathy which may cause macrosomia and subsequent mechanical complications during labor [17,18]. Additionally, studies suggest that children born to mothers with GDM have an increased risk of diabetes mellitus and metabolic dysfunction later in life [19,20]. In order to reduce the risk of such adverse health outcomes, current best practice GDM management requires modification of the maternal diet with or without pharmacological treatment such as Metformin and/or insulin [21]. Despite its benefits, pharmacotherapy may result in significant side effects including abdominal discomfort, dizziness, diarrhea and hypoglycemia [22]. As research suggests that probiotic interventions may attenuate some of the adverse metabolic effects of type 2 diabetes [23,24], probiotics may also provide an acceptable treatment option in women with GDM.

Probiotics have been defined by the World Health Organization (WHO) as ‘live micro-organisms which when administered in adequate amounts confer a health benefit on the host’ [25]. Regular consumption of probiotics have been found to beneficially modulate the composition of the gut microbiota [26]. Increases in colonic microbial diversity have been linked to improved glucose homeostasis, attenuation of inflammation, regulation of insulin production, maintenance of the integrity of the gastrointestinal lining, and the harvesting of nutrients from the host diet [7,8,27]. Safe and effective evidence-based interventions are vital for both the prevention and optimal management of GDM. A recent RCT conducted in healthy pregnant women suggest that probiotic supplementation may improve blood glucose control during the third trimester [28] and potentially reduce the risk of developing GDM [29]. To our knowledge, no systematic reviews have investigated the effect of maternal probiotic supplementation on the metabolic health of women with established GDM, highlighting the need for further exploration of this topic. The aim of this review was to determine the effect of 6–8 week probiotic supplementation versus placebo on glucose homeostasis, lipid levels and gestational weight gain in pregnant women diagnosed with GDM.

## 2. Methods

This review was conducted in accordance with the Preferred Reporting Items for Systematic Reviews and Meta-Analyses: The PRISMA Statement [30]. A systematic computer search of the databases Proquest, Scopus, Embase, Ovid MEDLINE, Web of science, CINAHL, and the Cochrane Database of Systematic Reviews was performed for the period between 1 January 2001 and 1 January 2017. In 2001, the WHO recognized the need for guidelines to evaluate probiotic use and to substantiate health claims. The rationale for excluding papers before 2001 was due to a lack of international guidelines and criteria regulating the use of probiotics prior to this date [31]. The following search terms were used: (1) (pregnan * OR Gestation * OR Matern * OR Obstetric * OR expectan * OR “gestational diabetes” OR “gestational diabetes mellitus”), (probiotic * OR Lactobacill * OR bacteria * OR ferment * OR microorganism * OR acidophilus OR streptococc *), and (glucose OR “blood glucose” OR insulin OR HbA1c OR “birth weight” OR metabol * OR intervention * OR “pharmaceutical Intervention”); (2) limit 1 to year ’2001–2017’; limit 2 to humans; limit 3 exclude males and non-pregnant subjects. Trials were included if they were published in English, utilized an RCT study design, involved human participants diagnosed with GDM by OGTT and if at least one group of participants were randomized to receive a dietary probiotic supplement for a period of 6–8 weeks.

Studies in patients with pre-existing conditions such as type 1 diabetes, type 2 diabetes or gastrointestinal pathologies were considered beyond the scope of this review, and were therefore excluded. Intervention trials involving the administration of fermented foods (which contain unknown quantities of bacteria), prebiotics (which contain no live bacteria) or synbiotics (which contain both pre- and probiotics) were also excluded.

Resultant studies were combined and duplicates removed. All articles were independently screened for eligibility by two authors based on title and abstract. Articles were excluded if they reported a non-RCT study design, subjects were not diagnosed with GDM or there was no probiotic intervention. The reference lists of included studies were hand-searched to identify additional relevant trials. The methodological quality of all included trials was independently assessed by two authors using the Cochrane Risk of Bias tool for Quality Assessment of Randomized Controlled Trials [32]. This tool rates primary research based on the use of sequence generation, allocation concealment, blinding of participants and personnel, blinding of outcome assessors, completeness of outcome data, non-selective outcome reporting, and other measures of bias. Trials were assessed as satisfying each of the quality criteria using “yes”, “no”, or “unsure”, with studies meeting the majority of quality criteria considered to have a low risk of bias, while those assigned “no” or “unsure” for most criteria were designated as moderate or high risk of bias. Discrepancies between authors risk of bias assessments were resolved through collaborative discussion until consensus was reached.

Data was independently extracted from each article by two authors using a data collection form. Data items collected included first author, article title, journal name, year of publication, country in which trial was conducted, number of trial participants: intervention group (*n*) and control group (*n*), mean participant age (year), mean participant BMI (kg/m^2^), mean gestational age (week), mean length of intervention (week), composition of probiotic supplement (genus, species), number of micro-organisms in probiotic supplement (CFU/g), mean gestational weight gain (kg), mean fasting blood glucose (mmol/L), mean Homeostasis Model Assessment—Insulin Resistance (HOMA-IR) (units), mean LDL-cholesterol (mmol/L), mean number of normal deliveries (*n*), mean number of caesarian sections (*n*), mean number of interventions during delivery (*n*), maternal complications (*n*), infant complications (*n*), and infant birth weight (g).

Trials measuring FBG, LDL-cholesterol and insulin resistance (HOMA-IR) in pregnant women with GDM were subjected to a random-effects model meta-analysis using Revman 5.1 (The Cochrane Collaboration, Copenhagen, Denmark, 2014). Treatment effects and 95% CI were calculated using the Mean Difference (MD). Limited numbers of studies investigating comparable outcomes, small sample sizes and heterogeneity among prebiotic supplements and outcome measures limited the majority of data synthesis to a narrative analysis.

## 3. Results

### 3.1. Description of Selected Trials

**Figure 1:**
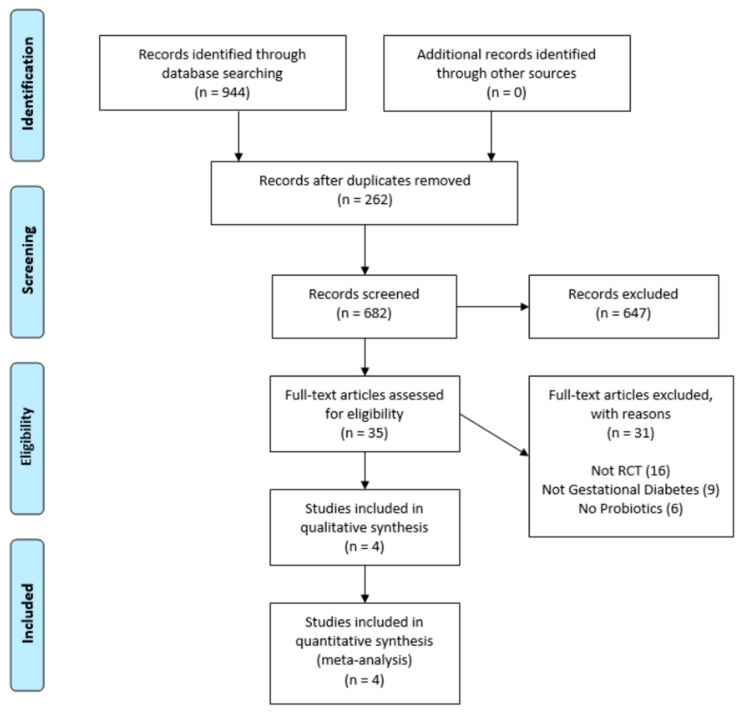
PRISMA flowchart showing the progression of trials through each stage of the selection process.

A total of 944 citations were identified at the time of the initial database search based on the predefined inclusion and exclusion criteria. After removal of duplicate publications and exclusion of irrelevant articles, four articles [33,34,35,36] reporting on four randomized controlled trials involving 288 participants were ultimately included (Figure 1). Characteristics of the included studies are shown in Table 1. All studies included otherwise healthy pregnant women diagnosed with GDM at 24–30 weeks gestation by oral glucose tolerance test. Participant ages ranged between 18–40 years and pre-pregnancy BMI from 26–32 kg/m^2^. All trial participants were randomized to receive either a daily probiotic supplement or a placebo. Probiotic composition varied between studies, but all trials provided *Lactobacillus* spp., and three [34,35,36] provided *Bifidobacterium* spp. The duration of intervention ranged from 6–8 weeks. A variety of post-intervention outcome measures were reported including fasting plasma glucose, fasting insulin, C-peptide, HOMA-IR, lipid studies, inflammatory markers, pro-inflammatory cytokines, gestational weight gain, requirement for glucose-lowering pharmacotherapy, interventions required during childbirth, infant birthweight, incidence of macrosomia (birthweight > 4 kg), fetal anomalies, admissions to the neonatal intensive care unit, and 5-min Apgar score.

All of the studies included in the present review had a low risk of bias, as assessed using the Cochrane Collaboration Risk of Bias tool (Table 2). Methodological strengths of the trials included double-blinding and randomization of participants to intervention and control groups. Methodological limitations of the trials included small sample sizes and short study duration. Additionally, one of the trials had not been registered on a clinical trials registry prior to commencement, so it could not be determined whether primary outcomes reported were pre-specified before the trial began [35].

### 3.2. Fasting Blood Glucose

**Figure 2:**
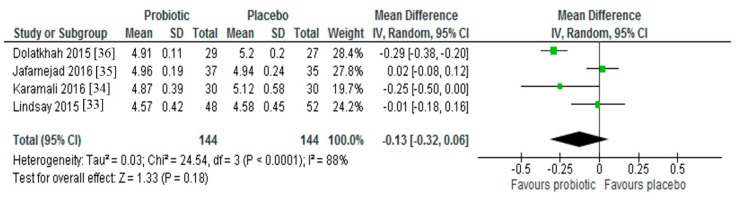
Effect of probiotic supplementation on fasting plasma glucose (mmol/L) in pregnant women with gestational diabetes.

Four studies investigated the effect of probiotic supplementation on FBG levels in pregnant women with GDM [32,33,34,35]. Two [33,35] of the four studies reported statistically significant reductions in FBG levels in the groups receiving probiotics in comparison to the groups receiving the placebo. However, a meta-analysis of all four trials (*n* = 288) indicated no significant reduction in FBG following probiotic supplementation (Mean Difference = −0.13; 95% CI −0.32, 0.06, *p* = 0.18) (Figure 2). While each of the studies included in the pooled analysis had a low risk of bias and administered probiotic supplements to women with GDM over a similar intervention period, significant interstudy heterogeneity was observed (*I*^2^ = 88%, *p* < 0.001), so the calculated mean difference should be interpreted as an average intervention effect.

### 3.3. Insulin Resistance

**Figure 3:**
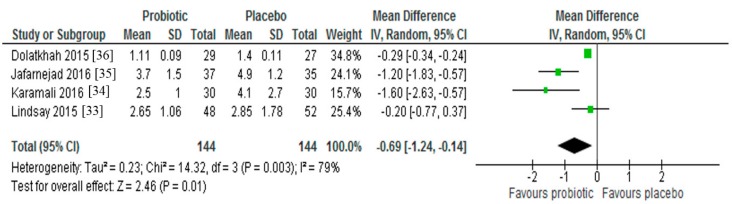
Effect of probiotic supplementation on HOMA-IR in pregnant women with gestational diabetes.

Four trials estimated insulin resistance in study participants by calculating HOMA-IR from fasting glucose and insulin values [33,34,35,36]. While one study found no change in insulin resistance between intervention and control groups following probiotic supplementation [33], three studies reported significant reductions in insulin resistance in the women receiving probiotics [34,35,36]. After meta-analysis (*n* = 288), the pooled mean difference in HOMA-IR was −0.69 (95% CI −1.24, −0.14, *p* = 0.01), indicating a statistically significant effect favoring probiotic supplementation over placebo (Figure 3). Significant evidence of interstudy heterogeneity was observed across studies (*I*^2^ = 79%, *p* < 0.01).

### 3.4. LDL-Cholesterol

**Figure 4:**
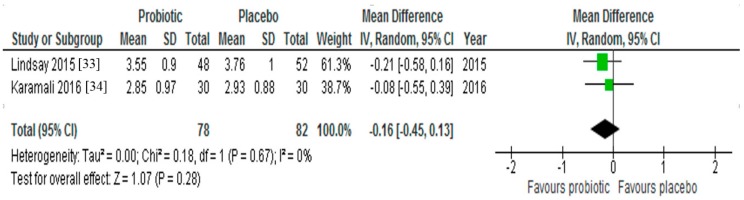
Effect of probiotic supplementation on LDL-cholesterol (mmol/L) in pregnant women with gestational diabetes.

Lindsay et al. [33] found that the usual rise in total cholesterol and LDL-cholesterol usually observed during the late stages of pregnancy was significantly attenuated in the probiotic group (both *p* < 0.05). In contrast, the study by Karamali et al. [34] reported no change in total cholesterol (*p* = 0.33) and LDL-cholesterol (*p* = 0.07) between treatment and control groups, but described significant reductions in VLDL-cholesterol and serum TG in the probiotic group (both < 0.05). When the data from both studies were pooled for meta-analysis (*n* = 160), there was no significant reduction in LDL-cholesterol following probiotic supplementation (MD = −0.16; 95% CI −0.45, 0.13, *p* = 0.67) (Figure 4).

### 3.5. Gestational Weight Gain

During the final 2 weeks of an 8-week intervention, one study reported that the weight gain of the women in the probiotic group was significantly less than the weight gain of those receiving the placebo (0.74 ± 0.14 kg vs. 1.22 ± 0.11 kg respectively), which remained significant after adjusting for daily energy intake (*p* < 0.05) [36]. However, the remaining three studies found no differences in gestational weight gain between intervention and control groups [33,34,35]. Two studies also reported no significant differences in infant birthweights between those born to mothers receiving the probiotic and those whose mothers received the placebo [33,34].

### 3.6. Obstetric Outcomes

No significant differences were found between probiotic and control groups for rates of pregnancy-induced hypertension, requirement for labor induction, commencement of glucose-lowering medications, blood loss at delivery, postpartum hemorrhage, fetal anomalies, admission of the infant to neonatal intensive care [33], and rates of delivery by caesarian section [33,34]. No adverse outcomes related to use of the probiotics were reported in any of the trials.

## 4. Discussion

Fasting hyperglycemia in women with GDM is associated with increased short and long-term morbidity in the offspring [37]. There is a clear need for safe, low-cost therapies to assist in the prevention and management of GDM. The gut microbial composition is altered during pregnancy, and given that specific micro-organisms in the gastrointestinal tract are able to positively influence host metabolism, probiotic supplements may contribute to the maintenance of bacterial diversity and glucose homeostasis in individuals with metabolic disturbances [38,39]. Research investigating probiotic use during pregnancy and its effect on the outcomes of GDM is an emerging area of interest. This systematic literature review aimed to explore the current evidence regarding the effect of probiotic supplementation on glucose and lipid homeostasis in pregnant women with GDM. Assessment of four randomized controlled trials in this review involving 288 pregnant women with GDM found that a 6–8 week probiotic intervention did not improve FBG or LDL-cholesterol levels. However, probiotic supplementation in women with GDM was associated with significant reductions in insulin resistance which could potentially reduce their requirement for glucose-lowering medication later in their pregnancy.

The mechanisms whereby probiotics alter glucose homeostasis are not completely understood. One proposed method is by the production of short chain fatty acids (SCFAs), generated as a by-product of bacterial fermentation of dietary fibers. SCFAs act as an energy source for intestinal cells and have been found to regulate the production of hormones effecting energy intake and expenditure such as leptin and grehlin [40]. The binding of SCFAs to G protein-coupled receptors GPR41 and GPR43 increases the intestinal expression of Peptide YY and Glucagon-like peptide-1 (GLP-1) hormones which act to reduce appetite by slowing intestinal transit time and increasing insulin sensitivity [11]. Another hypothesized mechanism of SCFA action includes reducing gastrointestinal permeability by upregulating transcription of tight junction proteins, enhancing production of Glucagon-like peptide-2 (GLP-2) which promotes crypt cell proliferation, and reducing inflammation in colonic epithelial cells by increasing PPAR-gamma activation [41]. Maintenance of the integrity of the gut barrier minimizes the concentration of lipopolysaccharide (LPS) in circulation. LPS is a structural component of gram negative bacterial cell walls, which induces an immune-cell response upon absorption into the human bloodstream, stimulating proinflammatory cytokine production and the onset of insulin resistance and hyperglycemia [42]. In support of this mechanism of probiotic action, Jafarnejad et al. [35] demonstrated probiotic-induced reductions in high sensitivity CRP, IL-6 and TNFα in their 8-week trial in women with GDM.

While this review found no significant effect of probiotic supplementation on FBG in women with GDM, a number of studies have reported positive outcomes. A study conducted in 256 pregnant women with normal glucose tolerance found significant reductions in FBG, insulin concentrations and insulin resistance following probiotic supplementation, potentially reducing participants’ GDM risk [28]. However, the length of the intervention (18 months) was significantly longer than the trials included in the current meta-analysis, and fasting glucose and insulin levels were measured during pregnancy and up to 12 months postpartum in the study subjects. A Cochrane systematic review exploring the effect of probiotic supplementation during normal pregnancies concluded that although there was a reduction in the incidence of GDM in one trial, there were insufficient studies to perform a quantitative meta-analysis [43]. Further research is therefore required before probiotics can be recommended to pregnant women to reduce their risk of GDM. A meta-analysis of six RCTs demonstrated a significant reduction in FBG in 252 subjects with type 2 diabetes [44], however changes in HbA1c, inflammatory markers, fasting insulin and HOMA-IR were inconclusive, possibly due to the brief duration of the intervention (4–8 weeks). It was also unknown whether trial participants were also receiving pharmacological therapy such as Metformin, which can influence the composition of the gut microbiota. The authors postulated that probiotics may elicit hypoglycemic effects by increasing the level of antioxidative enzymes capable of scavenging reactive oxygen species, thereby reducing oxidative stress levels [44]. Similarly, a systematic review of 12 RCTs explored the effect of probiotics on glucose tolerance in people with type 2 diabetes, concluding that probiotic supplementation significantly reduced FBG [45]. This review included trials which varied substantially in methodological quality, and a number of the probiotic treatments included yoghurts or other foodstuffs containing unknown quantities of uncertain bacterial species. Finally, a systematic review of 17 RCTs reported significant reductions in FBG, fasting insulin and HOMA-IR [46]. Trial participants represented a range of demographics with various forms of metabolic disease including GDM, hypercholesterolemia and T2DM [46], which was likely to have contributed to the large interstudy heterogeneity observed.

The contradictory findings of this review in comparison to other published reviews investigating the effect of probiotics on FBG may be related to the small sample sizes (*n* = 60–149) and short study durations (6–8 weeks) in the women with GDM. Moreover, the current review included trials involving only participants with GDM, which may be more resistant to the effects of probiotic supplementation than the variety of other forms of glucose intolerance included in the other reviews. Indeed, increased insulin resistance is considered a normal consequence of all healthy pregnancies [13]. As there is currently no consensus on the ideal bacterial composition and dose of probiotics for the management of glucose tolerance, the microbial components of the probiotics used in the GDM trials may not have been sufficient to effect FBG levels.

Two RCTs included in this review investigated the effect of probiotic supplementation on maternal lipid levels in GDM, with both reporting conflicting results [33,34]. While one study demonstrated that probiotic treatment may have mitigated the expected increase in total and LDL-cholesterol during pregnancy [33], the other trial reported significant reductions in VLDL-cholesterol and triglyceride, while a decrease in LDL-cholesterol approached significance [34]. Beneficial gut bacteria have been hypothesized to positively influence lipid metabolism by producing secondary bile acids which are unavailable for enterohepatic recirculation. The liver must then synthesize replacement bile acids from circulating cholesterol [47]. In the present review, a pooled analysis of LDL-cholesterol data from both studies in women with GDM was not significant. Trials of longer duration (>8 weeks) may have generated outcomes with larger effect sizes.

Three of the four studies included in this review [34,35,36] reported significant reductions in insulin resistance (as measured by HOMA-IR) following probiotic supplementation in women diagnosed with GDM. This did not appear to result in subsequent decreases in FBG, gestational weight gain or a reduced requirement for blood glucose-lowering medication in the intervention group, but further studies of longer duration should explore this. When all four studies were combined, there was a significant reduction in insulin resistance (MD = −0.69; 95% CI −1.24, −0.14, *p* = 0.01). The studies which found significant reductions in insulin resistance used *Bifidobacterium* spp. in their probiotic [34,35,36], whereas the study with non-significant findings did not [33]. *Bifidobacterium* spp. have been reported to play a protective role in the prevention of metabolic perturbations by reducing LPS-induced oxidative stress and low grade chronic inflammation [48].

The current management for GDM involves lifestyle changes through dietary modification and physical activity, and pharmacological intervention with metformin and/or insulin if required in order to achieve target blood glucose levels [49,50,51]. Metformin reduces hyperglycemia by increasing insulin sensitivity and reducing excessive hepatic gluconeogenesis [49]. Both insulin and metformin are considered safe for use in pregnancy, but can be associated with unwanted side effects such as gastrointestinal disturbances and hypoglycemia [52,53]. Metformin contributes to a healthy gut microbiome by increasing the growth of *Akkermansia muciniphila* and *Lactobacillus* spp. in murine studies, but further research in humans is required to confirm this [54]. Metformin-induced expansion of the *Akkermansia muciniphila* population has been shown to modulate glucose homeostasis in obese mice fed a high fat diet [55]. *Akkermansia muciniphilia* is a mucin-degrading bacterium important for the regulation of the thickness of the mucin layer lining the host gastrointestinal tract, thus protecting the integrity of the gut barrier [56]. Human studies are now required to determine whether metformin-induced improvements in gut microbial diversity contribute to improvements in glucose tolerance. The studies included in this review were not affected by participant use of metformin, as the women required to commence metformin or insulin during the course of the trials were excluded from the final analyses.

All of the RCTs in this review were determined to have a low risk of bias, with most authors publishing their study protocol in a clinical trials registry with pre-specified primary and secondary outcomes prior to study commencement. However, a limitation of these trials were their short duration and small sample sizes. Constraints associated with this systematic review include the substantial interstudy statistical heterogeneity observed, and the use of probiotics of differing microbial composition between trials. The metabolic benefits of probiotics may be strain specific, so the optimal species, dose and duration of treatment in GDM requires further elucidation.

## 5. Conclusions

The present review found that while probiotic supplementation resulted in a significant reduction in insulin resistance in pregnant women with GDM, there was no significant effect on fasting blood glucose or LDL-cholesterol levels. Further high quality studies using defined doses of specific bacterial species are required to confirm these findings and their clinical relevance before their routine use can be recommended in this patient group.

## Figures and Tables

**Table 1:** Characteristics of randomised controlled trials included in the review.

Study Author/Year	Participants	Study Design/Blinding	Dietary Probiotic Intervention	Effect of Dietary Probiotic Supplement on Metabolic Outcomes
Karamali et al. (2016) [34]	Iran, *n* 60 pregnant women with GDM in third trimester (age range 18–40 years)	Parallel RCT, double-blinded	Random assignment to 6-week probiotic or placebo capsules. Each probiotic capsule contained *L. acidophilus* (2 × 10^9^ CFU/g), *L. casei* (2 × 10^9^ CFU/g) and *B. bifidum* (2 × 10^9^ CFU/g)	↓ Fasting plasma glucose
				↓ HOMA-IR
				↔ Total cholesterol
				↔ LDL cholesterol
				↓ VLDL cholesterol
				↓ Triglyceride
				↔ gestational weight gain
Dolatkhah et al. (2015) [36]	Iran, *n* 64 pregnant women with GDM (mean age intervention 28.1 years, control 26.5 years; mean BMI intervention 31.4 kg/m^2^, control 29.9 kg/m^2^)	Parallel RCT, double-blinded	Random assignment to 8-week probiotic capsule with dietary advice or placebo capsule with dietary advice. Each probiotic capsule contained *L. acidophilus* LA-5, *Bifidobacterium* BB-12, *S.* thermophilus STY-31 and *L. delbrueckii* subsp. Bulgaricus LBY-27 (>4 × 10^9^ CFU/g)	↓ Fasting plasma glucose
				↓ HOMA-IR
				↓ gestational weight gain
Jafarnejad et al. (2016) [35]	Iran, *n* 82 pregnant women with GDM (mean age intervention 32.4 years control 31.9 years; mean BMI intervention 26.8 kg/m^2^, control 27.4 kg/m^2^)	Parallel RCT, double-blinded	Random assignment to 8-week probiotic or placebo capsules. Each probiotic capsule contained VSL#3 (*S. thermophilus*, *B. breve*, *B. longum*, *B. infantis*, *L. acidophilus*, *L. plantarum*, *L. paracasei*, *L. delbrueckii* subsp. Bulgaricus, 15 × 10^9^ CFU/g)	↓ Fasting plasma glucose
				↔ gestational weight gain
				↓ HOMA-IR
				↓ Interleukin-6
				↓ Tumor Necrosis Factor-aplha
				↓ hs-CRP
Lindsay et al. (2015) [33]	Ireland, *n* 149 women with GDM (mean age intervention 33.5 years control 32.6 years; mean BMI intervention 29.1 kg/m^2^, control 29.0 kg/m^2^)	Parallel RCT, double-blinded	Random assignment to 6-week probiotic or placebo capsules. Each capsule contained *L. salivarius* UCC118 (1 × 10^9^ CFU/g)	↔ Fasting plasma glucose
				↔ HOMA-IR
				↔ C-peptide
				↓ Total cholesterol
				↔ CRP
				↔ Triglyceride
				↓ LDL cholesterol
				↔ HDL cholesterol
				↔ gestational weight gain

RCT: Randomised Controlled Trial; GDM: Gestational Diabetes Mellitus; HOMA-IR score: homeostatic model of assessment of insulin resistance; hs-CRP: High sensitivity C-reactive protein; HDL cholesterol: High density lipoprotein; LDL cholesterol: Low density lipoprotein; HbA1c: Glycosylated haemoglobin; *n*: number of participants randomised; ↓ significantly lower than that in the comparison control group after intervention; ↑ significantly higher than that in the comparison control group after intervention; ↔ no significant difference between the probiotic-supplemented and control groups after intervention.

**Table 2:** Risk of bias summary for included studies.

Author/Year	Risk of Bias ^a^	Bias Minimisation Items ^b^						
		1	2	3	4	5	6	Other
Dolatkhah, 2015 [36]	Low	+	+	+	+	+	?	Funding & sponsorship free from bias, statistical analysis appropriate
Lindsay, 2015 [33]	Low	+	+	+	+	+	?	Funding & sponsorship free from bias
Jafarnejad, 2015 [35]	Low	+	+	+	+	?	?	Funding & sponsorship free from bias
Karamali, 2015 [34]	Low	+	+	+	+	+	?	Funding & sponsorship free from bias

“+” = response of “yes” to use of the bias minimization item; “?” = response of “uncertain” to the use of the bias minimization item; ^a^ Assessed using the Cochrane Collaboration tool for assessing risk of bias in RCTs (ref); ^b^ Bias minimization items: 1. Random sequence generation (selection bias); 2. Allocation concealment (selection bias); 3. Blinding of participants and personnel (performance bias); 4. Blinding of outcome assessment (detection bias); 5. Complete outcome data (attrition bias); 6. Non-selective reporting (reporting bias). Trials receiving a + response for most items are likely to have a low risk of bias.
